# Ethanol Infusion of Vein of Marshall for the Treatment of Persistent Atrial Fibrillation: The Basics and Clinical Practice

**DOI:** 10.3390/jcdd9080270

**Published:** 2022-08-16

**Authors:** Bo He, Fang Zhao, Wenxi Yu, Yi Li, Xiaoyan Wu, Zhibing Lu

**Affiliations:** 1 Department of Cardiology, Zhongnan Hospital of Wuhan University, 169 Donghu Road, Wuhan 430071, China; 2 Cardiovascular Institute, Zhongnan Hospital of Wuhan University, Wuhan 430071, China; 3 Institute of Myocardial Injury and Repair, Wuhan University, Wuhan 430071, China

**Keywords:** persistent atrial fibrillation, vein of Marshall, perimitral flutter, ethanol infusion, epicardial connection

## Abstract

Catheter ablation for persistent atrial fibrillation (PeAF) is particularly challenging, as the clinical outcomes are modest. Pulmonary vein isolation (PVI) plus linear ablation is one of the main strategies for PeAF ablation. Completely durable transmural lesions are difficult to achieve by catheter ablation during mitral isthmus ablation. The ligament of Marshall contains the vein of Marshall (VOM), myocardial tracts and innervation, and serves as arrhythmogenic foci that make it an attractive target in catheter ablation of atrial fibrillation. Additionally, it co-localizes with the mitral isthmus, and may serve as a part of the perimitral isthmus reentrant circuit. Ethanol infusion into the VOM results in rapid ablation of the neighboring myocardium and its innervation. Its incorporation into PVI significantly increases the success rate of mitral isthmus block and the clinical outcome of PeAF ablation.

## 1. Introduction

Catheter ablation for persistent atrial fibrillation (PeAF) is particularly challenging as the clinical outcomes are modest [1], which may be associated with the progressive nature of atrial fibrillation (AF). Pulmonary vein isolation (PVI) remains the cornerstone of PeAF ablation [2,3]. In an European survey study, stand-alone PVI was performed as a first-time ablation for patients with PeAF in two-thirds of the surveyed centers [4]. Besides PVI, other ablation strategies, including the ablation of complex fractionated electrograms, placement of linear lesions, stepwise approach until AF termination, and substrate modification of low-voltage areas, are also applied in PeAF ablation. As shown in the STAR AF II study [5], no reduction in the recurrence of AF was achieved when either linear ablation or the ablation of complex fractionated electrograms was performed in addition to PVI. Although it seems that substrate modification following PVI adds no further benefits to PVI alone, recent studies have indicated that PVI plus linear ablation and ethanol infusion into the vein of Marshall (VOM) in PeAF exhibits a favorable outcome [6,7]. PVI with linear ablation, as one of the main strategies for PeAF ablation, mimics the Cox maze procedure using completely blocked lines to prevent the formation of reentrant circuits. It has been shown that the freedom from AF after the Cox maze III procedure at a follow-up of more than 5 years could be up to 97% [8]. However, this could not be repeated by catheter ablation. Two reasons may explain the different results. Firstly, catheter ablation using a linear ablation approach does not create as many lines as the Cox maze III procedure dose. The Cox maze III procedure creates blocked lines by incisions in the right atrium, which is not usually performed during catheter ablation. Secondly, completely durably transmural lesions are difficult to create by catheter ablation, especially when discrete anatomical structures, such as the coronary sinus (CS) and its branches, especially the VOM, have epicardial muscular bundles that serve as a component of the reentrant circuits [9,10,11,12,13]. Recently, VOM ethanol infusion, which aims to improve the outcome of PeAF ablation by targeting the VOM or the ligament of Marshall (LOM), has been the focus of attention of clinical electrophysiologists. This review focuses on the basics and clinical practice of VOM ethanol infusion in PeAF ablation.

## 2. Anatomy and Electrophysiological Characteristics of the LOM/VOM

**Figure 1:**
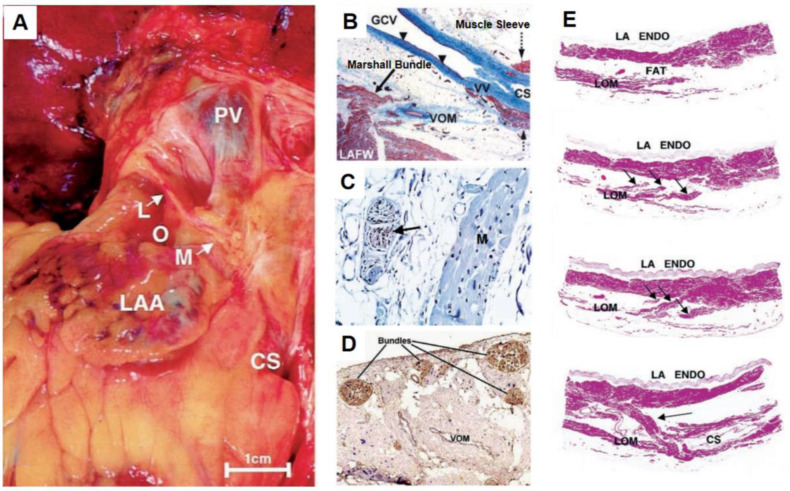
Gross photo (**A**) and histochemical staining (**B**–**E**) showing the anatomy of the ligament of Marshall. (**B**) The CS myocardial sleeve is shown by Masson’s trichrome staining of the CS and VOM, which ends at the valve of Vieussens (VV). No myocardial sleeve is seen in the great cardiac vein (GCV). (**C**) The nerve (brown staining-arrow) is shown by positive avidin-biotin-peroxidase immunohistochemical staining for tyrosine. (×120). (**D**) Dual-staining of the nerve bundles of the LOM for DBH (blue) and ChAT (brown) showing that cholinergic nerve fibers are predominant (×5). (**E**) Hematoxylin-eosin staining showing the tracts of the LOM. Three tracts (arrows) emerging from the LOM are shown by subserial sections, which eventually enter the atrial wall. The last panel shows a section from the lower end of the LOM. The tract inserts into the left atrial wall (arrow) and CS (×10). CS, coronary sinus; ChAT, choline acetyl transferase; DBH, dopamine β-hydroxylase; LAA, left atrial appendage; LAFW, left atrial free wall; LOM, ligament of Marshall; M, myocardium; F, fat; PV, pulmonary vein; VOM, vein of Marshall (reproduced and modified with permission from Kim et al. [15], Chou et al. [16], and Ulphani et al. [17]).

The LOM is located on the epicardial aspect of the ridge between the left atrial appendage and left pulmonary veins. The LOM is a remnant of the embryonic sinus venosus and left cardinal vein, which contains fat and fibrous tissues, blood vessels (VOM), muscle bundles, nerve fibers, and ganglia [14,15,16] (Figure 1). As an important component of the LOM, the VOM drains the posterior and posterolateral wall of the left atrium (LA) and runs obliquely and inferiorly to the CS. It drains directly into the CS, with its ostium proximal to the Vieussens valve.

The LOM is richly innervated by both cholinergic and adrenergic nerves and ganglion cells. Different portions of the LOM present different cholinergic-to-adrenergic innervation ratios [18]. The PV-LA junctions are densely covered by tyrosine hydroxylase-stained sympathetic nerve fibers. However, non-tyrosine hydroxylase-stained parasympathetic ganglions are dominant at the CS juncture. A progressive increase in the parasympathetic ganglions and a progressive decrease in the sympathetic nerve fibers are observed from the distal portion (the PV-LA junctions) to the proximal portion (the CS juncture) of the LOM. These differences were also verified by an electrophysiological study, in which high-frequency stimulation of the proximal portion of the LOM preferentially induced AF, while stimulation of the distal portion mainly induced ventricular arrhythmias [19].

**Figure 2:**
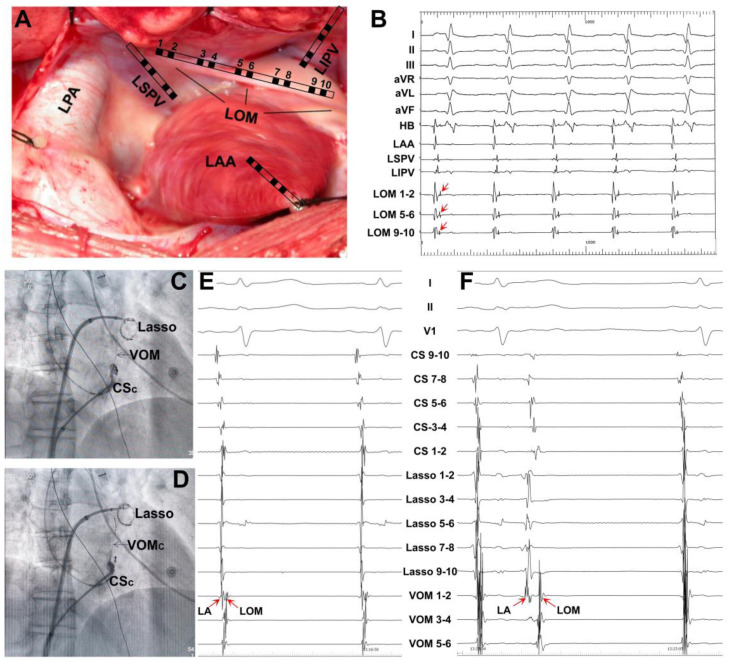
Recordings of the potentials of the ligament of Marshall (LOM) in dogs and humans. (**A**) The catheter position and anatomy of LOM in a dog. (**B**) Simultaneous recording of surface ECG lead I, II, III, aVR, aVL, aVF, HB, LAA, LSPV, LIPV, LOM_1-2_, LOM_5-6_, and LOM_9-10_ in a dog showing LOM potentials (arrows). (**C**,**D**) Vein of Marshall (VOM) venography and LOM potentials recorded with a small catheter (VOMc) placed in the VOM in a patient with persistent atrial fibrillation. The Lasso catheter is positioned in the LSPV. (E,F) Simultaneous recording of surface ECG lead I, II, V_1_, CS_1-2_~CS_9-10_, Lasso_1-2_~Lasso_9-10_, and VOM_1-2_~VOM_5-6_. During a sinus beat, the left atrium potential (LA) is fused with the LOM potential (**E**). A premature atrial beat separates the LA and the LOM potentials (**F**). HB, His bundle electrogram; LAA, left atrial appendage; LSPV, left superior pulmonary vein; LIPV, left inferior pulmonary vein; CSc, coronary sinus catheter.

Scherlag et al. [20] first recorded double potentials (the left atrial muscle potential and the LOM potential) along the LOM in canines. The LOM was considered insulated from the left atrial muscle, as an equipotential line exists between these two potentials. The explanation would be that the LA is activated via the Bachman bundle with a faster conduction velocity, but the LOM is activated by the CS conduction with a slower speed during sinus rhythm, producing separated double potentials. In our study [21], we recorded separated LOM potentials in 28/32 dogs (Figure 2) and fused electrograms in 4/32 dogs, respectively. The activation sequence in the LOM was from proximal to distal during sinus beats, with the LOM potential following the LA potential. Usually, the LA potential can be separated from the LOM potential by rapid atrial pacing. Occasionally, triple potentials, i.e., the LA potential, the PV potential, and the LOM potential, can be recorded near the left superior PV. A thin catheter can be inserted into the VOM to record the LOM potential in patients (Figure 2).

## 3. The LOM/VOM and AF: Experimental Findings

Several arrhythmogenic mechanisms of the LOM have been proposed. Firstly, as a source of dual sympathetic and parasympathetic innervation, early afterdepolarization-induced triggered firing could produce ectopic beats that could initiate atrial tachyarrhythmias. Secondly, the complexity of the LOM facilitates the formation of localized microreentry, driving atrial tachyarrhythmias. Thirdly, the electrical conduction properties of the LOM, as well as the epicardial connection to the atrial tissue, make the LOM serve as a component of macroreentrant circuits.

Scherlag et al. [20] first provided proof of the LOM as an arrhythmogenic source. In dogs, an ectopic rapid atrial rhythm originating from the LOM could be successfully induced by stimulating the left cardiac sympathetic nerves. In a normal canine atrium, Doshi et al. [22] also demonstrated the LOM as an origin site of isoproterenol-sensitive (adrenergic) focal atrial tachycardia. Kim et al. [15] further provided anatomical evidence for the arrhythmogenic role of LOM by studying seven postmortem human hearts. The LOM was found to consist of multiple myocardial tracts directly inserted into the left atrial free wall and CS, involving the substrate of reentry. The change in the myocardial bundle’s effective refractory period induced by the activation of sympathetic nerves in the LOM could promote the initiation and perpetuation of atrial tachycardia. During atrial pacing, the high-frequency stimulation of the LOM could successfully induce AF, and this induction could be inhibited by both esmolol and atropine [19,23]. In addition, LOM ablation significantly prolonged the effective refractory period of the tissues near the LOM and reduced AF inducibility [24]. All of these findings demonstrated that both parasympathetic and sympathetic nerves play a vital role in AF [25,26]. Calcium transient is amplified during sympathetic activation by the increases in calcium influx and calcium release from the sarcoplasmic reticulum, resulting in higher intracellular calcium concentrations during phase 3 repolarization. A pause caused by parasympathetic activation further enhances calcium transient. A net inward current created by the Na^+^/Ca^2+^ exchanger, which extrudes one calcium ion in exchange for importing three sodium ions, could produce early afterdepolarizations and trigger firing [26]. Additionally, the shortened action potential duration and effective refractory period induced by parasympathetic activation promote the initiation and perpetuation of AF. The left superior PV is the most common triggering site for AF and is anatomically close to the distal portion of the LOM. Kamanul et al. [27] demonstrated a direct electrical connection between the left superior PV and LOM. The electrical coupling between the LOM and left superior PV may contribute to rapid firing within the left superior PV during AF.

In a short-term rapid atrial pacing canine model, we investigated the effects of ablating the distal portion of the LOM on atrial electrical remodeling [28]. In the dogs that received rapid atrial pacing first, the shortened atrial effective refractory period induced by rapid atrial pacing was reversed by LOM ablation. LOM ablation also reduced AF inducibility. However, neither the change in the atrial effective refractory period nor AF were induced by rapid atrial pacing in the dogs that received LOM ablation first. The sympathetic indices of heart rate variability, as well as serum norepinephrine concentration, were decreased. These results indicated that the distal portion of LOM may be responsible for sympathetic AF and may serve as a potential AF ablation target.

## 4. The LOM/VOM and AF: Clinical Evidence

Rapid atrial activity recorded from the VOM indicated the VOM/LOM as a source of AF [27,29]. In patients with sustained AF, Kamanu et al. [29] recorded the ectopic activity from the VOM with a complex local electrogram, and found that the activation rate of VOM ectopy was significantly faster than those of other atrial and PV sites. Spectral analysis showed that the dominant frequency at the VOM was significantly higher than that at other atrial sites. Catheter ablation of the insertion site of the VOM successfully terminated AF and prevented its reinduction in four of these six patients [27]. Similar findings were reported by Valderrabano et al. [30,31]. In a cohort of patients receiving catheter ablation of AF or postablation of atrial tachycardia, 18 patients (32%) had a LOM-PV connection, and a LOM-mediated atrial tachycardia was present in 13 patients (23%) [10]. Thirty-one patients with refractory mitral isthmus (MI) conduction received ethanol infusion into the VOM, which completely isolated the left-sided PVs and left atrial appendage, or slowed or terminated the perimitral reentry. These findings confirm the participation of the VOM/LOM in the initiation and maintenance of AF and atrial tachycardia. The VOM also plays a role in PV reconnection in AF recurrence. Dave et al. [32] demonstrated that VOM ethanol infusion could eliminate these left inferior PV and left superior PV reconnections in 23/32 and 13/30 patients, respectively.

## 5. LOM/VOM as Epicardial Connection Mediates Perimitral Flutter after AF Ablation

**Figure 3:**
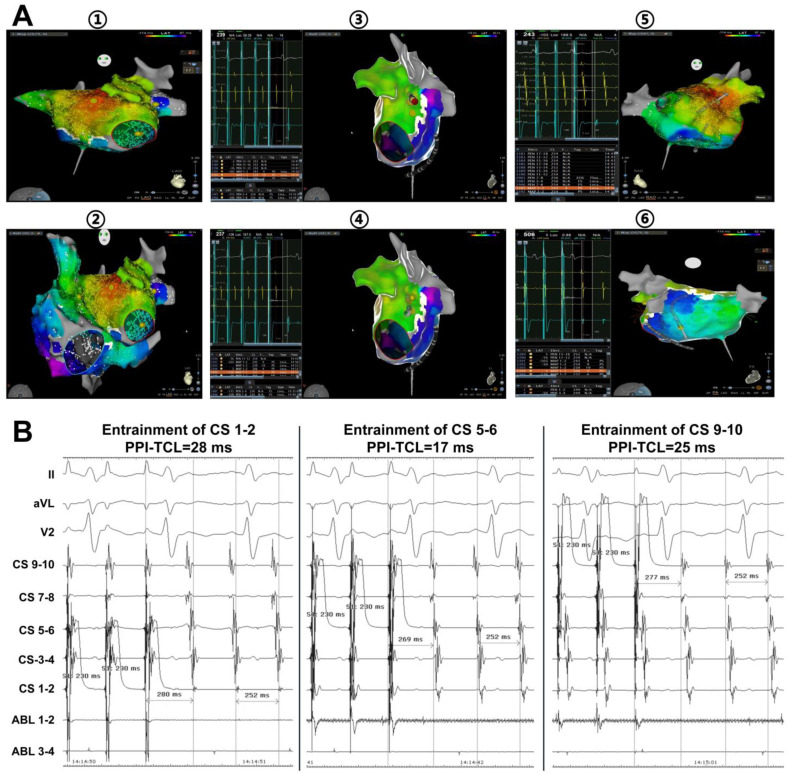

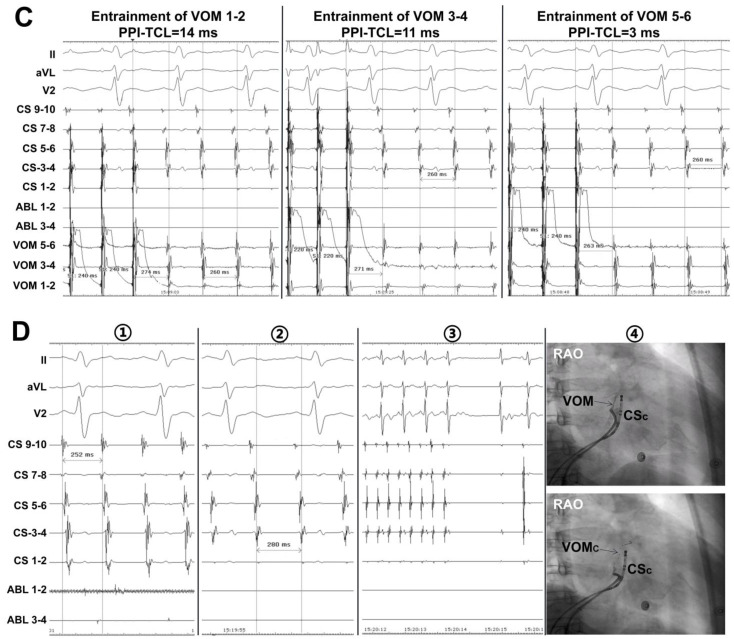
Perimitral flutter mediated by VOM epicardial connections in a persistent atrial fibrillation patient undergoing PVI and linear ablation. (**A**) Activation mapping of the left atrium (①) and right atrium (②), and entrainment mapping of the left atrium (③～⑥). Activation mapping of the left atrium indicated a focal activation mode, and the earliest atrial activation was located at the anterior roof. The total activation time of the left atrium was 201 ms, less than the TCL of 252 ms. Activation mapping of the right atrium showed that the right atrium was passively activated by the left atrium. Entrainment from MI (③ and ④) produced significantly prolonged PPI (PPI-TCL = 72 ms in ③; PPI-TCL = 82 ms in ④), while entrainment from the anterior roof (⑤) and posterior inferior wall near the CS (⑥) produced short PPIs (PPI-TCL = 12 ms in ⑤; PPI-TCL = 7 ms in ⑥). (**B**) Entrainment from CS produced short PPIs. (**C**) Entrainment from VOM also produced short PPIs. The activation mapping and entrainment mapping indicated that the tachycardia was MI-dependent with an epicardial connection (VOM). (**D**) Selective VOM venography prolonged the TCL without terminating the tachycardia (① and ②). Injection of 2 mL of ethanol into VOM terminated the tachycardia (③). Fluoroscopic images of a VOM venogram and VOM catheter (VOMc) were shown in right anterior oblique (RAO) view (④). PVI, pulmonary vein isolation; VOM, vein of Marshall; TCL, tachycardia cycle length; MI, mitral isthmus; PPI, postpacing interval; CS, coronary sinus.

As an epicardial connection, the LOM/VOM more commonly mediates macro-reentrant AT, especially perimitral flutter, in patients undergoing AF ablation (Figure 3). In a cohort of 240 patients with symptomatic AF who received a single-ring PVI procedure, Chik et al. [33] reported five tachycardias involving the LOM region. Low voltage, long-duration fractionated potentials, or mid-diastolic potentials were recorded during tachycardia in the LOM region, which was remote from the endocardial breakout site. The ablation of these potentials successfully eliminated tachycardia. Hayashi et al. [34] reported that the reentrant circuits related to LOM accounted for up to 11% of perimitral ATs following PVI or valve surgery. Vlachos et al. [9] also showed that Marshall bundle reentrant ATs accounted for up to 30.2% of the left ATs after AF ablation. The bidirectional block of either the Marshall bundle-LA or CS-Marshall bundle connections was required to eliminate these perimitral ATs.

## 6. The Challenge and Importance of MI Block

MI line ablation is usually performed in PeAF ablation when a linear ablation strategy is applied. The confirmation of complete MI block is a very important step during mapping and ablation procedures. It has been shown that MI block improves the outcome of PeAF ablation [35]. Conventionally, MI block was evaluated by comparing the trans-isthmus conduction time and the CS activation sequence during septal pacing and lateral pacing, respectively. If complete MI block is achieved, counterclockwise activation around the mitral annulus will be seen during left atrial appendage pacing [36], while a prolonged trans-isthmus conduction time (from the pacing CS electrode to the left atrial appendage) will also be seen during distal CS pacing when compared with that during proximal CS pacing [37]. However, the reversal of the CS activation sequence during left atrial appendage pacing and the prolongation of the trans-isthmus conduction time during distal CS pacing may be insufficient to confirm complete MI block if epicardial conduction or a gap with very slow conduction exists. Barkagan et al. [12] reported that 21.6% of patients in whom MI block was confirmed by pacing had residual endocardial or epicardial connections during activation mapping. A distance of 2.4 ± 1.6 cm was observed between the epicardial bridging connections and the MI line. The insertion site was located septally at the left atrial ridge and laterally at the proximal-middle CS. Epicardial connections were more commonly seen in patients with residual conduction. MI line block was achieved in 3/4 of the patients when ablating the insertion sites. By using both the VOM and CS electrodes, Fujisawa et al. [38] found that pseudo-block of MI could be seen in 1/3 of the patients undergoing MI ablation.

The difficulty in achieving complete MI block could be due to the limited lesion induced by the radiofrequency energy and the complicated anatomy of the MI. Firstly, the length and thickness of MI will be variable in patients. Even in a patient, the thickness of MI in different regions is not even. Thus, a fixed ablation index in the MI line may not produce transmural lesions in certain parts of the MI. Secondly, the blood flow in the CS and the circumflex artery will take away part of the energy, making the transmural lesion insufficiently formed. Thirdly, epicardial connections from the CS and/or VOM are out of reach of endocardial radiofrequency energy applications.

## 7. The Role of VOM Ethanol Infusion in PeAF Ablation

Multiple effects can be achieved by VOM ethanol infusion. First, ectopic triggers in VOM can be successfully eliminated by ethanol infusion. Second, the anterior wall of the left pulmonary veins, as well as the ridge between the left atrial appendage and the left pulmonary veins, can be sufficiently damaged, increasing the durability of PVI and modifying the substrate in MI. Third, residual conduction involving the epicardial connections mediated by VOM, which may produce complex perimitral circuits, can be effectively blocked, reducing the occurrence of perimitral atrial flutter after PeAF ablation. All these together would reduce AF recurrence after PeAF ablation. In a cohort of 75 PeAF patients with PVI, VOM ethanol infusion, and mitral, roof, and cavotricuspid isthmus ablation, a 79% success rate was observed in patients undergoing a single procedure, while an 89% success rate was shown in patients undergoing one or two procedures in 12 months [6]. In another cohort of 191 consecutive patients with PeAF, a higher success rate (87.9%) was observed in patients with linear ablation and VOM ethanol infusion following PVI compared with patients with PVI plus linear ablation (64.8%) during a 12-month follow-up [7]. Liu et al. [39] reported that nonparoxysmal AF patients who received substrate modification and VOM ethanol infusion following PVI had better long-term freedom from AF and atrial arrhythmia compared with patients who received PVI alone or with substrate modification during a follow-up of 3.9 ± 0.5 years. VOM ethanol infusion was the independent predictor of freedom from AF recurrence and atrial arrhythmia during multivariate analysis. The randomized clinical trial VENUS included 343 patients with PeAF who were randomly assigned to catheter ablation alone (n = 158) or catheter ablation combined with VOM ethanol infusion (n = 185). A significantly higher freedom rate from AF or prolonged atrial tachycardia (49% vs. 38% at both 6 and 12 months) was observed in patients who received catheter ablation with VOM ethanol infusion [40]. In patients with PeAF who underwent a second procedure, the strategy using systematic linear ablation with VOM ethanol infusion had lower recurrence when compared with the strategy using atrial tachyarrhythmia termination as the procedural endpoint [41]. A recent meta-analysis including 1337 patients compared the long-term outcomes between catheter ablation with VOM ethanol infusion and ablation alone [42]. The combination procedure provided significantly better outcomes than ablation alone, while the safety of both procedures was comparable. Additionally, in patients who underwent redo procedures after previous VOM ethanol infusion for PeAF or perimitral LA flutter, electroanatomical mapping showed that the VOM ethanol infusion-related lesion was durable [43]. The clinical studies of EIVOM in PeAF are summarized in Table 1. Although emerging clinical evidence indicates superior efficacy of VOM ethanol infusion following catheter ablation in PeAF, VOM ethanol infusion has been not universally applied in PeAF ablation. Thus, more randomized controlled trials with large sample sizes are required to further validate the role of VOM ethanol infusion in PeAF ablation.

## 8. Technique for VOM Ethanol Infusion

**Figure 4:**
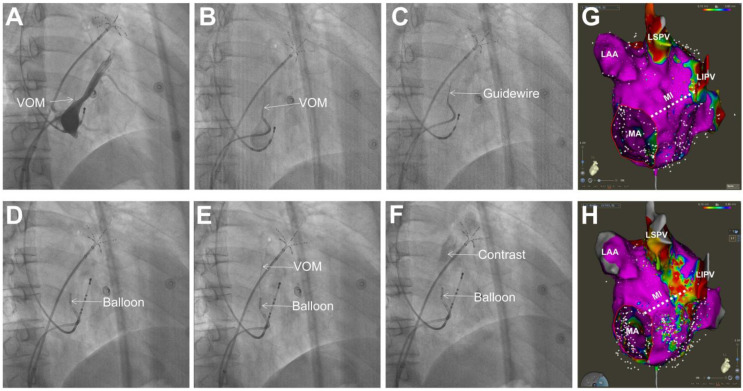
Fluoroscopic images in RAO view showing the procedural steps of VOM ethanol infusion. (**A**) Coronary sinus venography showing the presence of VOM. (**B**) Selective VOM venography. (**C**) Guidewire advanced into the VOM. (**D**) Balloon advanced into the VOM and inflated. (**E**) Selective VOM venography via the wire port of the balloon performed to confirm complete occlusion. (**F**) Contrast injection into VOM showing myocardial staining with the contrast medium after ethanol infusion. (**G**) Voltage mapping of posterior MI, left pulmonary vein-LAA ridge region, and left pulmonary veins before VOM ethanol infusion. (**H**) Voltage mapping in the same region showing low-voltage regions in the anatomical distribution of the VOM after VOM ethanol infusion. LAA, left atrial appendage; LSPV, left superior pulmonary vein; LIPV, left inferior pulmonary vein; MI, mitral isthmus; MA, mitral annulus. RAO, right anterior oblique.

VOM ethanol infusion can be considered in PeAF patients if MI line ablation is included in catheter ablation, especially in patients who had previous failed catheter ablation due to MI-dependent flutter or suspected CS/VOM-mediated macroreentrant tachycardia. Retrograde balloon cannulation of the VOM from the coronary sinus is feasible and allows for ethanol delivery, which results in the rapid ablation of the neighboring myocardium and its innervation. In an experienced center, VOM cannulation and ethanol infusion of up to 89% can be achieved [46,47]. VOM ethanol infusion failure mainly resulted from the nonidentification of the VOM, noncannulation of the VOM, or ethanol infusion inside an incorrect vein [46]. Valderrabano et al. [30,31] firstly introduced the procedural steps for the retrograde infusion of ethanol in the VOM. In our experience, several key steps are included (Figure 4). First, a 6-F guiding catheter is introduced from the 8.5-F-long sheath via the right femoral vein into the CS, and a CS venogram is performed by direct contrast injection through the guiding catheter in the right anterior oblique view to assess the presence or absence of the VOM. Second, selective VOM venography is performed by the cannulation of the VOM using the same guiding catheter to further show the size and the course of VOM. Third, an angioplasty guidewire (Runthrough™ guidewire, Terumo, Tokyo, Japan) is advanced into the VOM as far as possible to secure cannulation. Fourth, an appropriately sized angioplasty balloon (1.5–2.5 mm diameter) is advanced into the distal VOM and inflated at 4–6 atm pressure, and the guidewire is then removed. Fifth, selective VOM venography is then performed to confirm complete occlusion by 0.5 mL contrast medium injection from the wire port of the balloon. Sixth, 2 mL of 98% ethanol is delivered during balloon inflation. The ethanol infusion repeated every 2 min. A total of 4–6 mL of ethanol will be injected into the distal VOM. After ethanol infusion finishes in the distal VOM, the balloon will be drawn back to the proximal VOM. Another 4–6 mL of ethanol will be injected into the proximal VOM. During ethanol infusion, repeated VOM venography can be performed to verify the integrity of the vein. Up to 10–12 mL of ethanol can be used in each patient.

## 9. The Limitations of VOM Ethanol Infusion

In terms of the limitations of VOM ethanol infusion, we should be cautious about its utility in PeAF ablation. Firstly, the anatomical course of the VOM determines the lesion sites and VOM ethanol infusion areas [48]. Based on this, the mitral annular side of the MI is usually beyond the lesion region of VOM ethanol infusion (Figure 4G,H). Thus, additional radiofrequency applications after VOM ethanol infusion will be needed to achieve MI block. Although MI line ablation with VOM ethanol infusion significantly reduces the time and radiofrequency applications needed to achieve MI block, the success rate of MI block and the reconduction rate in patients with radiofrequency ablation alone are similar to those in patients with the combination procedure [49]. Moreover, VOM is not the only epicardial connection of MI. Epicardial musculature related to the great cardiac vein has been shown to serve as important residual MI gaps after VOM ethanol infusion [11]. These gaps are usually eliminated by radiofrequency applications within the great cardiac vein.

## 10. Conclusions

Both experimental studies and clinical trials demonstrate the arrhythmogenic role of LOM/VOM in AF. LOM/VOM profoundly contributes to left atrium macro-reentrant circuits, especially perimitral flutter in patients with prior catheter ablation of AF. VOM ethanol infusion is feasible and safe, and achieves rapid ablation of LA tissue and local innervation. Adding VOM ethanol infusion to catheter ablation significantly improves the outcome of PeAF ablation while further confirmatory trials are under way.

## Figures and Tables

**Table 1:** Summary of clinical studies of EIVOM in PeAF.

Reference	Study Type	Study Population	Ablation Strategy		Primary Endpoint	N		Total Procedure Time (min)	Follow-Up Duration	Complications	Key Findings
			EIVOM	Control		EIVOM	Control				
Pambrun et al. 2019 [44]	Single-arm	Persistent AF	EIVOM + PVI + MI line + Roof line + CTI	-	Freedom from arrhythmia recurrence	10	-	270 ± 29.9	6 m	None	AF termination and noninducibility were achieved in 50% and 90% of the patients, respectively. All patients were free from arrhythmia recurrence during follow-up.
Liu et al. 2019 [39]	Retrospective, observational	Non-paroxysmal AF	PVI + substrate modification + EIVOM	PVI + substrate modification or PVI	A recurrence of AF or any atrial arrhythmia	32	64 and 32	125.4 ± 65.6 vs. 149.7 ± 45.9 vs. 113.4 ± 52.1	3.9 ± 0.5 y	-	Total atrial arrhythmia recurrence was 28.1%, 59.7%, and 44.6%, respectively. Left atrial diameter >45 mm and hypertension were independent risk factors for recurrence.
Valderrábano et al. 2020 [40]	Multi-center randomized controlled trial	Persistent AF	Catheter ablation (sequential approach) + EIVOM	Catheter ablation alone	Freedom from AF or AT after a single procedure, without AADs, at both 6 and 12 months	185	158	215.9 ± 77.7 vs. 190.3 ± 63.5	6 m and 12 m	34/185 vs. 27/158	Freedom from AF or tachycardia was 49.2% (91/185) vs. 38% (60/158) at 6 and 12 months after a single procedure. After multiple procedures, freedom from AF was 65.2% vs. 53.8%.
Nakashima et al. 2020 [45]	Retrospective, observational, single-center	Persistent AF (>97%)	EIVOM + PVI+ MI ablation + additional substrate modification	PVI+ MI ablation + additional substrate modification	12-month freedom from AF/AT/AFL	152	110	276 ± 60 vs. 263 ± 69	291 ± 170 d	2/152 vs. 1/110	During follow-up, 31.6% (48/152) of patients in the EIVOM group and 75.5% (83/110) of patients in the RFCA group experienced recurrent AF or AT, respectively. Acute and durable MI blocks were more frequently achieved in the EIVOM group.
Derval et al. 2021 [6]	Prospective, observational, single-arm	Persistent AF	EIVOM + PVI + MI line + Roof line + CTI	-	12-month freedom from AF/AT without AADs	75	-	277 ± 41	12 m	transient ischemic attack (2), postablation pericarditis (4), minor groin hematomas (3)	At 12 months, 72% (54/75) and 89% (67/75) of patients were free from AF/AT after a single procedure or 1 or 2 procedures, respectively.
Lai et al. 2021 [7]	Prospective, observational, single-center	Persistent AF	EIVOM + PVI + MI line + Roof line + CTI	PVI + MI line + Roof line + CTI	Free from AF/AT at 12 months	66	125	162.4 ± 39.7 vs. 171.5 ± 44.8	12 m	3% vs. 5.6%	At 12 months, 58/66 (87.9%) patients in EIVOM group and 81/125 (64.8%) patients in control group were free from AF/AT, respectively.
Nakashima et al. 2022 [41]	Retrospective, observational, single-center	Persistent AF with a previous failed ablation	EIVOM + PVI + MI line + Roof line + CTI	PVI + ablation of complex atrial activities + linear ablation	Free from AF/AT at 12 months	96	102	222 ± 57 vs. 267 ± 93	12 m	0/96 vs. 3/102	At one-year follow-up, 21/96 (22%) patients in EIVOM group and 38/102 (37%) patients in control group had AF/AT recurrence, respectively.

PVI, pulmonary vein isolation; EIVOM, ethanol infusion of vein of Marshall; PeAF, Persistent AF; AF, atrial fibrillation; AT, atrial tachycardia; AFL, atrial flutter; MI, mitral isthmus; CTI, cavotricuspid isthmus; AAD, anti-arrhythmic drugs.

## References

[1] Schreiber D., Rostock T., Fröhlich M., Sultan A., Servatius H., Hoffmann B.A., Lüker J., Berner I., Schäffer B., Wegscheider K. (2015). Five year follow-up after catheter ablation of persistent atrial fibrillation using the stepwise approach and prognostic factors for success. Circ. Arrhythm. Electrophysiol..

[2] Calkins H., Hindricks G., Cappato R., Kim Y.H., Saad E.B., Aguinaga L., Akar J.G., Badhwar V., Brugada J., Camm J. (2018). 2017 HRS/EHRA/ECAS/APHRS/SOLAECE expert consensus statement on catheter and surgical ablation of atrial fibrillation. Europace.

[3] Hindricks G., Potpara T., Dagres N., Arbelo E., Bax J.J., Blomström-Lundqvist C., Boriani G., Castella M., Dan G.A., Dilaveris P.E. (2021). 2020 ESC Guidelines for the diagnosis and management of atrial fibrillation developed in collaboration with the European Association for Cardio-Thoracic Surgery (EACTS): The Task Force for the diagnosis and management of atrial fibrillation of the European Society of Cardiology (ESC) Developed with the special contribution of the European Heart Rhythm Association (EHRA) of the ESC. Eur. Heart J..

[4] Dagres N., Bongiorni M.G., Larsen T.B., Hernandez-Madrid A., Pison L., Blomstrom-Lundqvist C. (2015). Scientific Initiatives Committee, European Heart Rhythm Association. Current ablation techniques for persistent atrial fibrillation: Results of the European Heart Rhythm Association Survey. Europace.

[5] Verma A., Jiang C.Y., Betts T.R., Chen J., Deisenhofer I., Mantovan R., Macle L., Morillo C.A., Haverkamp W., Weerasooriya R. (2015). Approaches to catheter ablation for persistent atrial fibrillation. N. Engl. J. Med..

[6] Derval N., Duchateau J., Denis A., Ramirez F.D., Mahida S., André C., Krisai P., Nakatani Y., Kitamura T., Takigawa M. (2021). Marshall bundle elimination, Pulmonary vein isolation, and Line completion for ANatomical ablation of persistent atrial fibrillation (Marshall-PLAN): Prospective, single-center study. Heart Rhythm.

[7] Lai Y., Liu X., Sang C., Long D., Li M., Ge W., Liu X., Lu Z., Guo Q., Jiang C. (2021). Effectiveness of ethanol infusion into the vein of Marshall combined with a fixed anatomical ablation strategy (the “upgraded 2C3L” approach) for catheter ablation of persistent atrial fibrillation. J. Cardiovasc. Electrophysiol..

[8] Prasad S.M., Maniar H.S., Camillo C.J., Schuessler R.B., Boineau J.P., Sundt T.M., Cox J.L., Damiano R.J. (2003). The Cox maze III procedure for atrial fibrillation: Long-term efficacy in patients undergoing lone versus concomitant procedures. J. Thorac. Cardiovasc. Surg..

[9] Vlachos K., Denis A., Takigawa M., Kitamura T., Martin C.A., Frontera A., Martin R., Bazoukis G., Bourier F., Cheniti G. (2019). The role of Marshall bundle epicardial connections in atrial tachycardias after atrial fibrillation ablation. Heart Rhythm.

[10] Chugh A., Gurm H.S., Krishnasamy K., Saeed M., Lohawijarn W., Hornsby K., Cunnane R., Ghanbari H., Latchamsetty R., Crawford T. (2018). Spectrum of atrial arrhythmias using the ligament of Marshall in patients with atrial fibrillation. Heart Rhythm.

[11] Pambrun T., Derval N., Duchateau J., Denis A., Chauvel R., Tixier R., Welte N., André C., Nakashima T., Nakatani Y. (2021). Epicardial course of the musculature related to the great cardiac vein: Anatomical considerations and clinical implications for mitral isthmus block after vein of Marshall ethanol infusion. Heart Rhythm.

[12] Barkagan M., Shapira-Daniels A., Leshem E., Shen C., Anter E. (2019). Pseudoblock of the Posterior Mitral Line with Epicardial Bridging Connections Is a Frequent Cause of Complex Perimitral Tachycardias. Circ. Arrhythm. Electrophysiol..

[13] Sakamoto Y., Lockwood D., Yamaguchi R., Yoshimoto D., Suzuki T., Ho S.Y., Nakagawa H. (2021). Systematic Evaluation of High-Resolution Activation Mapping to Identify Residual Endocardial and Epicardial Conduction Across the Mitral Isthmus. JACC Clin. Electrophysiol..

[14] Marshall J. (1850). On the development of the great anterior veins in man and mammalia: Including an account of certain remnants of foetal structure found in the adult, a comparative view of these great veins in the different mammalia, and an analysis of their occasional peculiarities in the human subject. Philos. Trans. R. Soc. Lond..

[15] Kim D.T., Lai A.C., Hwang C., Fan L.-T., Karagueuzian H.S., Chen P.-S., Fishbein M.C. (2000). The ligament of Marshall: A structural analysis in human hearts with implications for atrial arrhythmia. J. Am. Coll. Cardiol..

[16] Chou C.C., Kim D.T., Fishbein M.C., Chen P.S. (2003). Marshall bundle and the valve of vieussens. J. Cardiovasc. Electrophysiol..

[17] Ulphani J.S., Arora R., Cain J.H., Villuendas R., Shen S., Gordon D., Inderyas F., Harvey L.A., Morris A., Goldberger J.J. (2007). The ligament of Marshall as a parasympathetic conduit. Am. J. Physiol. Heart Circ. Physiol..

[18] Makino M., Inoue S., Matsuyama T.A., Ogawa G., Sakai T., Kobayashi Y.-I., Katagiri T., Ota H. (2006). Diverse myocardial extension and autonomic innervation on ligament of marshall in humans. J. Cardiovasc. Electrophysiol..

[19] Lin J., Scherlag B.J., Lu Z., Zhang Y., Liu S., Patterson E., Jackman W.M., Lazzara R., Po S.S. (2008). Inducibility of atrial and ventricular arrhythmias along the ligament of marshall: Role of autonomic factors. J. Cardiovasc. Electrophysiol..

[20] Scherlag B.J., Yeh B.K., Robinson M.J. (1972). Inferior interatrial pathway in the dog. Circ. Res..

[21] Wang S., Lu Z., He W., Xie J., Yu X., Jiang H. (2016). Selective Ablation of the Ligament of Marshall Reduces the Prevalence of Ventricular Arrhythmias Through Autonomic Modulation in a Cesium-Induced Long QT Canine Model. JACC Clin. Electrophysiol..

[22] Doshi R.N., Wu T.J., Yashima M., Kim Y.-H., Ong J.J.C., Cao J.-M., Hwang C., Yashar P., Fishbein M.C., Karagueuzian H.S. (1999). Relation between ligament of Marshall and adrenergic atrial tachyarrhythmia. Circulation.

[23] Lin J., Scherlag B.J., Niu G., Lu Z., Patterson E., Liu S., Lazzara R., Jackman W.M., Po S.S. (2009). Autonomic elements within the ligament of Marshall and inferior left ganglionated plexus mediate functions of the atrial neural network. J. Cardiovasc. Electrophysiol..

[24] Liu X., Yan Q., Li H., Tian Y., Su J., Tang R., Lu C., Dong J., Ma C. (2010). Ablation of ligament of Marshall attenuates atrial vulnerability to fibrillation induced by inferior left atrial fat pad stimulation in dogs. J. Cardiovasc. Electrophysiol..

[25] Patterson E., Po S.S., Scherlag B.J., Lazzara R. (2005). Triggered firing in pulmonary veins initiated by in vitro autonomic nerve stimulation. Heart Rhythm.

[26] Patterson E., Lazzara R., Szabo B., Liu H., Tang D., Li Y.-H., Scherlag B.J., Po S.S. (2006). Sodium-calcium exchange initiated by the Ca^2+^ transient: An arrhythmia trigger within pulmonary veins. J. Am. Coll. Cardiol..

[27] Kamanu S., Tan A.Y., Peter C.T., Hwang C., Chen P.S. (2006). Vein of Marshall activity during sustained atrial fibrillation. J. Cardiovasc. Electrophysiol..

[28] Yu X., He W., Qin Z., Liu S., Ma R., Luo D., Hu H., Xie J., He B., Lu Z. (2018). Selective ablation of the ligament of Marshall attenuates atrial electrical remodeling in a short-term rapid atrial pacing canine model. J. Cardiovasc. Electrophysiol..

[29] Hwang C., Wu T.J., Doshi R.N., Peter C.T., Chen P.S. (2000). Vein of Marshall cannulation for the analysis of electrical activity in patients with focal atrial fifibrillation. Circulation.

[30] Valderrábano M., Liu X., Sasaridis C., Sidhu J., Little S., Khoury D.S. (2009). Ethanol infusion in the vein of Marshall: Adjunctive effects during ablation of atrial fibrillation. Heart Rhythm.

[31] Valderrábano M., Chen H.R., Sidhu J., Rao L., Ling Y., Khoury D.S. (2009). Retrograde ethanol infusion in the vein of Marshall: Regional left atrial ablation, vagal denervation and feasibility in humans. Circ. Arrhythm. Electrophysiol..

[32] Dave A.S., Báez-Escudero J.L., Sasaridis C., Hong T.E., Rami T., Valderrábano M. (2012). Role of the vein of Marshall in atrial fibrillation recurrences after catheter ablation: Therapeutic effect of ethanol infusion. J. Cardiovasc. Electrophysiol..

[33] Chik W.W., Chan J.K., Ross D.L., Wagstaff J., Kizana E., Thiagalingam A., Kovoor P., Thomas S.P. (2014). Atrial tachycardias utilizing the Ligament of Marshall region following single ring pulmonary vein isolation for atrial fibrillation. Pacing Clin. Electrophysiol..

[34] Hayashi T., Fukamizu S., Mitsuhashi T., Kitamura T., Aoyama Y., Hojo R., Sugawara Y., Sakurada H., Hiraoka M., Fujita H. (2016). Peri-Mitral Atrial Tachycardia Using the Marshall Bundle Epicardial Connections. JACC Clin. Electrophysiol..

[35] Lador A., Peterson L.E., Swarup V., Schurmann P.A., Makkar A., Doshi R.N., DeLurgio D., Athill C.A., Ellenbogen K.A., Natale A. (2021). Determinants of outcome impact of vein of Marshall ethanol infusion when added to catheter ablation of persistent atrial fibrillation: A secondary analysis of the VENUS randomized clinical trial. Heart Rhythm.

[36] Jaïs P., Hocini M., Hsu L.F., Sanders P., Scavee C., Weerasooriya R., Macle L., Raybaud F., Garrigue S., Shah D.C. (2004). Technique and results of linear ablation at the mitral isthmus. Circulation.

[37] Shah A.J., Pascale P., Miyazaki S., Liu X., Roten L., Derval N., Jadidi A.S., Scherr D., Wilton S.B., Pedersen M. (2012). Prevalence and types of pitfall in the assessment of mitral isthmus linear conduction block. Circ. Arrhythm. Electrophysiol..

[38] Fujisawa T., Kimura T., Nakajima K., Nishiyama T., Katsumata Y., Aizawa Y., Fukuda K., Takatsuki S. (2019). Importance of the vein of Marshall involvement in mitral isthmus ablation. Pacing Clin. Electrophysiol..

[39] Liu C.M., Lo L.W., Lin Y.J., Lin C., Chang S., Chung F., Chao T., Hu Y., Tuan T., Liao J. (2019). Long-term efficacy and safety of adjunctive ethanol infusion into the vein of Marshall during catheter ablation for nonparoxysmal atrial fibrillation. J. Cardiovasc. Electrophysiol..

[40] Valderrábano M., Peterson L.E., Swarup V., Schurmann P.A., Makkar A., Doshi R.N., DeLurgio D., Athill C.A., Ellenbogen K.A., Natale A. (2020). Effect of Catheter Ablation with Vein of Marshall Ethanol Infusion vs. Catheter Ablation Alone on Persistent Atrial Fibrillation: The VENUS Randomized Clinical Trial. JAMA.

[41] Nakashima T., Pambrun T., Vlachos K., Goujeau C., André C., Krisai P., Ramirez F.D., Pintican G., Kamakura T., Takagi T. (2022). Strategy for repeat procedures in patients with persistent atrial fibrillation: Systematic linear ablation with adjunctive ethanol infusion into the vein of Marshall versus electrophysiology-guided ablation. J. Cardiovasc. Electrophysiol..

[42] Li F., Sun J.Y., Wu L.D., Zhang L., Qu Q., Wang C., Qian L.L., Wang R.X. (2022). The Long-Term Outcomes of Ablation with Vein of Marshall Ethanol Infusion vs. Ablation Alone in Patients With Atrial Fibrillation: A Meta-Analysis. Front. Cardiovasc. Med..

[43] Laredo M., Ferchaud V., Thomas O., Moubarak G., Cauchemez B., Zhao A. (2022). Durability of Left Atrial Lesions After Ethanol Infusion in the Vein of Marshall. JACC Clin. Electrophysiol..

[44] Pambrun T., Denis A., Duchateau J., Sacher F., Hocini M., Jaïs P., Haïssaguerre M., Derval N. (2019). MARSHALL bundles elimination, Pulmonary veins isolation and Lines completion for ANatomical ablation of persistent atrial fibrillation: MARSHALL-PLAN case series. J. Cardiovasc. Electrophysiol..

[45] Nakashima T., Pambrun T., Vlachos K., Goujeau C., André C., Krisai P., Ramirez F.D., Kamakura T., Takagi T., Nakatani Y. (2020). Impact of Vein of Marshall Ethanol Infusion on Mitral Isthmus Block: Efficacy and Durability. Circ. Arrhythm. Electrophysiol..

[46] Kamakura T., Derval N., Duchateau J., Denis A., Nakashima T., Takagi T., Ramirez F.D., André C., Krisai P., Nakatani Y. (2021). Vein of Marshall Ethanol Infusion: Feasibility, Pitfalls, and Complications in Over 700 Patients. Circ. Arrhythm. Electrophysiol..

[47] Rodríguez-Mañero M., Schurmann P., Valderrábano M. (2016). Ligament and vein of Marshall: A therapeutic opportunity in atrial fibrillation. Heart Rhythm.

[48] Kamakura T., André C., Duchateau J., Nakashima T., Nakatani Y., Takagi T., Krisai P., Ascione C., Balbo C., Tixier R. (2022). Distribution of atrial low voltage induced by vein of Marshall ethanol infusion. J. Cardiovasc. Electrophysiol..

[49] Ishimura M., Yamamoto M., Himi T., Kobayashi Y. (2021). Durability of mitral isthmus ablation with and without ethanol infusion in the vein of Marshall. J. Cardiovasc. Electrophysiol..

